# Cancellation of elective surgery and associated factors among patients scheduled for elective surgeries in public hospitals in Harari regional state, Eastern Ethiopia

**DOI:** 10.3389/fmed.2023.1036393

**Published:** 2023-04-04

**Authors:** Damte Adugna, Teshager Worku, Ahmed Hiko, Merga Dheresa, Shiferaw Letta, Addisu Sertsu, Haregeweyn Kibret

**Affiliations:** School of Nursing and Midwifery, College of Health Medical Sciences, Haramaya University, Harar, Ethiopia

**Keywords:** cancellation, elective surgery, operation theatre, Harar, Ethiopia

## Abstract

**Background:**

Canceling elective surgeries is a significant problem in many hospitals leading to patient dissatisfaction, increased costs, and emotional trauma for patients and their families. Despite this, there is limited information about the cancellation of elective surgeries in Ethiopia, mainly in the study area.

**Objective:**

This study aimed to assess the magnitude of cancellation and associated factors among patients scheduled for elective surgeries in public hospitals in the Harari Regional State, Eastern Ethiopia, from 1 August to 30 August 2021.

**Methods:**

A hospital-based cross-sectional study was conducted on 378 patients scheduled for elective surgeries. Data were gathered using a non-random sequential sampling approach. In addition, a structured face-to-face interviewer-administered questionnaire was employed. The gathered information was input into Epidata version 3.1 and then exported to Statistical Package for Social Software version 26. To find the variables associated with the cancellation of elective surgeries, binary and multi-variable logistic regression analyses were conducted. In the binary analysis, all variables with a *p*-value of less than 0.25 were included in the multivariable analysis. Finally, a 0.05 *p*-value with a 95% confidence interval and an adjusted odds ratio was used to declare a significant association.

**Results:**

This study included 378 patients scheduled for elective surgeries. Among those, 35.2% of the surgeries were canceled (95% confidence interval: 29.4–39.6). Being female (adjusted odds ratio: 2.46; 95% confidence interval: 1.44–4.203), lack of formal education (adjusted odds ratio: 2.03; 95% confidence interval: 1.15–3.58), place of residence (adjusted odds ratio: 1.70; 95% confidence interval: 1.03–2.81), increase in blood pressure (adjusted odds ratio: 5.09; 95% confidence interval:1.90–13.59), and ophthalmologic surgery (adjusted odds ratio: 3.76; 95% confidence interval: 1.41–10.0) were factors associated with the cancellation of elective surgeries.

**Conclusion:**

In this study, nearly one third of scheduled elective surgery was canceled. The primary contributing variables to the surgery cancellations were being female, lack of formal education, place of residence, ophthalmologic surgery, and increased blood pressure. Therefore, timely evidence-based reporting through the supervision team was advised to decrease cancellations.

## Introduction

An elective surgical case cancellation is when an operation is planned but not carried out as scheduled (1). Previous research showed that the prevalence of surgery cancellations ranged from 1.9 to 49% (2, 3). The cancellation rate surpasses 20% in wealthy nations (4). However, among less-developed nations, the percentage is 48.5%, with Ethiopia at 33.9% (3, 5).

The cancellation of elective surgery is a problem with the healthcare system’s quality that impacts individuals and wastes resources. Particularly, it depresses the spirits of workers, patients, and family members, which may result in lower levels of efficiency at work (6). Most research divided cancellation into avoidable and unavoidable factors (7–10). According to studies, just 20% of cancellations were inevitable, while more than 80% might have been avoided (8, 11, 12).

Various nations and hospitals have different reasons for canceling elective surgical procedures (6). Because they include patients and hospital operational difficulties, the causes of elective surgical case cancellations are complicated (6, 13). Psychological, social, and financial factors, not just clinical ones, were the basis for delaying surgeries after admission. Cancellations of elective surgeries can frustrate patients, tax hospital resources, waste money on medical supplies, and cause other inefficiencies in medical treatment (5). An important goal for a healthcare system is to increase patient satisfaction *via* effective practice. It is challenging to do this due to the high cancellation rate for elective surgical treatments. In addition, cancellation lowers operating room productivity and raises expenditures (14, 15).

In clinical practice, canceling elective surgical procedures are quite prevalent in Ethiopia. The majority of earlier research projects in Ethiopia were restricted to determining the number and severity of reasons for postponing elective surgeries. They could only provide a proportional breakdown of the causes and reasons for postponing elective surgeries (7, 10, 13). In general, there is little information available on the cancellation of elective procedures in the eastern part of Ethiopia, despite the significance of the issue. Therefore, the purpose of this study was to evaluate the extent and contributing variables of elective surgical procedure cancellation in public hospitals in the Harari Region, Eastern Ethiopia.

## Materials and methods

### Study design and setting

A hospital-based cross-sectional study was conducted in the public health facilities of Harar city, Eastern Ethiopia, from 1 August to 30 August 2021. Harar is the capital city of the Harari regional state, which is 526 km away from Addis Ababa. According to the Ethiopian Central Statistical Agency’s estimated Population Projections for 2015–2045, the Harari region will have a total population of 220,000 by 2021, of which 111,000 (50.46%) of them are men (16). There are 19 kebeles in Harar town. In the Harari region, there are 26 health posts, eight health centers, and six hospitals (four public and two private). Among those public hospitals, Hiwot Fana Specialized University Hospital (HFSUH) and Jugal General Hospital provide healthcare services to people living around Harar and neighboring regions such as Oromia Regional State, Dire Dawa Administration, and Somali Regional State (17).

### Populations and sampling procedure

All age groups of patients admitted to the surgical ward, the gynecology ward, the pediatric ward, and the ophthalmology ward throughout the study period and were scheduled for elective surgeries were included in the study. Both registered planned patients for elective surgery transferred to other institutions and patients enrolled for elective surgery but had it done as an emergency before the appointed date were excluded. By considering the following assumptions, the sample size was calculated using the single population formula: 95% confidence interval, 5% margin of error, and 33.9% prevalence of canceled elective surgery (5). The sample size for this study was obtained by adding a 10% non-response rate, and the final sample size was 378.

### Data collection tools and methods

The questionnaires were developed by reviewing previous studies in a way that included all the variables that could help the study achieve its goals (5, 7, 10, 13). It was finally conceived in terms of the local setting. The questionnaire was prepared in English and translated into the local language Afan Oromo to collect information from the study participants. The tool is divided into three sections: socio-demographic factors (age, sex, educational level, and place of residence), procedure-related factors (general, orthopedic, gynecology, pediatric, and ophthalmology), and cancellation-related variables (patient refusal, abnormal lab investigation, patients unfit for anesthesia, shortage of surgical equipment, lack of oxygen, overscheduled cases, etc. During data collection, the medical records of patients were reviewed to identify the types of procedures performed, whether elective surgical procedures were performed or not, the types of diagnoses, the reason for cancellation, and so on. A structured face-to-face interviewer-administered questionnaire was also used to gather the data. Again we used a medical record review and a telephone survey to gather relevant information from those who were slated for elective surgery but were missing on the appointed date once they were permitted to go home for financial preparation for surgery. Four skilled BSc nurses were recruited outside of study settings to gather the data, and two MSc nurses functioned as supervisors. All COVID-19 safety measures were followed over the whole data collection period.

### Variables and measurement

The study’s dependent variable was the cancellation of surgical cases. Age, Sex, Departments of Patient Admission, and Patients Related factors (Medical condition, Refusal, absence) facility-related factors (lack of ICU bed, Scarcity of equipment, lack of electricity); time constraints (prolonged case, overscheduling); personnel-related factors (surgeon, anesthetist, nurse); incomplete investigation were Independent Variables. A planned surgery that is not carried out on the scheduled day is referred to as a surgical procedure cancellation. Non-emergency surgery that may be postponed for at least 24 h is known as elective surgery.

### Data quality assurance

The data collectors were chosen from outside the research site to minimize bias. Data collectors and supervisors received 1 day of training on the data collecting instruments, research objectives, methods of data collection, ethical issues, and a brief explanation to ensure common comprehension. Before the actual data collection period, a pilot test on the data collection instruments was conducted at Haramaya General Hospital on 5% of the sample size to guarantee validity. Later, significant feedback were included in the questionnaire. Supervisors and the principal investigator afterward reviewed the information gathered; any issues or questions were promptly resolved.

### Statistical analysis

The data were manually reviewed for completeness after data collection, then they were cleaned, coded, and entered using Epi-Data 3.1, and lastly, they were exported to SPSS version 26 for analysis. Summary measures from texts, tables, and graphs were used to describe the findings of the descriptive statistical study.

Using the variance inflation factor (VIF) and standard error, multicollinearity was examined to see whether there was a linear association between the linked independent variables. VIF values of more than 10 or standard errors greater than 2 were seen as indicators of multicollinearity. Because of this, it was determined that variables having a VIF >10 or a standard error >2 should not be included in the multivariable analysis. But in our investigation, no variable’s VIF or standard error exceeded 10. In bivariable analysis, those factors with a *p*-value < 0.25 at a 95% confidence interval were transferred to multivariable analysis. A multivariable logistic regression model using the Enter method was employed in the multivariate analysis to adjust the confounders. To assess the model’s fitness, the Hosmer–Lemeshow goodness-of-fit test and the Omnibus test were conducted. As a result, in this investigation, the Hosmer–Lemeshow’s test was found to be insignificant at *p*-value = 0.567 and the Omnibus test was significant at *p*-value = 0.0001, indicating that the model was fitted. The adjusted odds ratio (AOR), with 95% confidence intervals (CI), was used to identify the independent variables associated with the dependent variable. A *P*-value of 0.05 was used to determine statistical significance, which excludes the null value from the 95% confidence range.

## Results

### Participants’ socio-demographic profiles

The research comprised 378 people who were scheduled for elective surgical procedures. The subjects’ mean (± SD) age was 33.98 (± 12.65). One hundred fifty (39.7%) were 20–49 years old, 225 (59.5%) were rural inhabitants, and 218 (57.7%) were men. Furthermore, 229 (60.6%) of the total respondents had no formal education, while 123 (32.5%) worked as farmers (Table 1).

**TABLE 1:** Socio-demographic characteristics of participants scheduled for elective surgical procedures in public hospitals in Harari regional state, Eastern Ethiopia, 2021 (*n* = 378).

Variable	Category	Frequency	Percent (%)
Age	<10	76	20.1
	10–19	50	13.2
	20–49	150	39.7
	≥50	102	27.0
Gender	Male	218	57.7
	Female	160	42.3
Educational status	No formal education	229	60.6
	Primary	63	16.7
	Secondary	43	11.4
	Preparatory	16	4.2
	College and above	27	7.1
Place of residence	Rural	225	59.5
	Urban	153	40.5
Occupation	Farmer	123	32.5
	Government employee	22	5.8
	Private employee	63	16.7
	Merchant	17	4.5
	Housewife family	65	17.2
	Others	88	23.3

### The magnitude of canceled elective surgery

**FIGURE 1:**
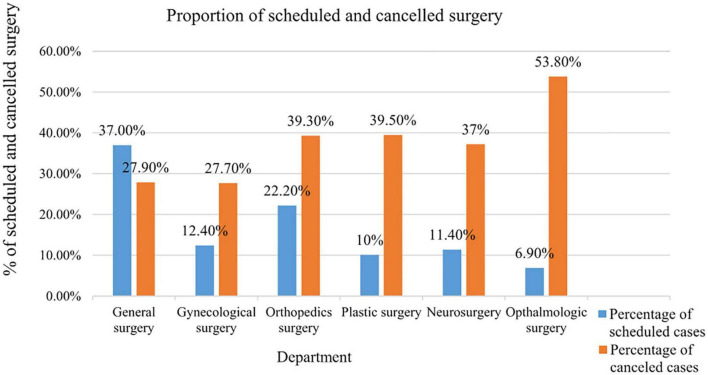
Proportion of scheduled and canceled surgery by the department in public hospitals in Harari regional state, Eastern Ethiopia, 2021 (*n* = 378).

In this analysis, the overall magnitude of elective surgery cancellation was 133 (35.2%) (95% CI: 29.4–39.6). Of this, 130 (97.7%) were canceled before entering the operating room (OR). Around 140 (37.0%) patients were scheduled for general surgery, with 84 (22.22% of patients) scheduled for orthopedic surgery. In the current survey, more than half of all respondents (53.8%) canceled elective surgery from Ophthalmologic surgery, followed by plastic surgery (39.5%) as indicated in the Figure 1.

### Reasons for cancellation

From a total of 133 patients, the administration-related case was the most common (42.6%), and the patient-related case was the second most common (40%) reason for cancellation of elective surgery.

Administration-related reasons for cancellation of elective surgery included blood not being prepared (11.3%), lack of laboratory investigations (7.5%), and a 5.3% power outage.

The most common patient-related reasons for elective case cancellation were patient refusal (13.5%), absence (9%), and 16.5% of medical disease-related cancellations of elective surgery were attributable to patients’ rising blood pressure as indicated in Table 2.

**TABLE 2:** Reported reasons for cancellation of elective surgery in public hospitals in Harari regional state, Eastern Ethiopia (*n* = 133).

Categories	Variables	Frequency (*n* = 133)	Percentages (%)
Patients	Patient refusal	18	13.5
	Not follow preoperative instruction	10	7.5
	Absenteeism	12	9
Medical disease	A rise in BP/HTN	22	16.5
	Rise in RBS/DM or acute medical illness	8	6
Facility/administrative	Lack of important laboratory investigation	10	7.5
	Shortage of OR materials	6	4.5
	Power breakdown	7	5.3
	Lack of ICU bed	4	3
	Blood not prepared	15	11.3
Professional	Surgeon	8	6
	Anesthesia provider	7	5.3
Shortage of time	Unexpected/case load	6	4.5

### Factors associated with the cancellation of elective surgery

In the binary logistic regression, sex, age, respondents’ educational status, place of residence, department, time scheduled for surgery, patient refusal, comorbidity, and blood not prepared were all related to the cancellation of elective surgery. To control the effect of confounding variables, the variables having a *p-value* of less than 0.25 were chosen for multi-variable logistic regression analysis. However, in a multivariate logistic regression analysis, only sex, place of residence, educational status, department, and comorbidity were significantly associated with the cancellation of elective surgery at a *p-value* of less than 0.05.

In multiple logistic regression, females were 2.46 times more likely than men to have elective surgery cancellation (AOR = 2.46, 95% CI: 1.44–4.20, *p* = 0.002). Individuals from the rural residences had 1.70 times the chance of canceling elective surgery as compared to participants from urban areas (AOR = 1.70, 95% CI: 1.03–2.81, *p* = 0.004). Those with no formal education had a 2.03 times greater chance of having elective surgery cancellation (AOR = 2.03, 95% CI: 1.15–3.58, *p* = 0.045) than those with formal education. Participants with higher blood pressure were 5.09 times more likely than their peers to cancel elective surgery (AOR = 5.09, 95% CI: 1.90–13.59, *p* = 0.009). Finally, individuals in the department of ophthalmologic surgery were 3.76 times more likely to have elective surgery cancellation than those in other departments (AOR = 3.76, 95% CI: 1.41–10.00, *p* = 0.01) with a *p*-value of 0.05, as shown below (Table 3).

**TABLE 3:** Bivariate and multi-variable logistic regression analysis of factors associated with the cancellation of elective surgery in public hospitals in Harari regional state, Eastern Ethiopia (*n* = 378).

Category		Yes % cancellation	COR (95% CI)	AOR (95% CI)
**Sex**				
	Female	70 (45.00)	2.05 (1.33–3.15)*	2.46 (1.44–4.203)**
	Male	60 (27.98)	1	
**Age, in year**				
	0–9	29 (38.2)	1	1
	10–19	5 (10.0)	0.18 (0.51_0.06)	0.29 (0.08–1.07)
	20–49	68 (45.3)	1.34 (2.36_0.77)	2.50 (0.93–6.70)
	≥50	28 (27.5)	0.61 (1.16_0.33)	0.96 (0.37–2.52)
**Place of residence**				
	Rural	89 (39.6)	1.79 (1.14–2.79)	1.70 (1.03–2.81)**
	Urban	41 (26.8)	1	1
**Educational status**				
	Illiterate	85 (31.0%)	1.63 (1.05–2.53) *	2.03 (1.15–3.58)**
	Literate	45 (28.1)	1	1
**Time scheduled**				
	Afternoon	64 (38.3	1.37 (0.89–2.09)*	1.45 (0.89–2.36)
	Morning	66 (31.3)	1	1
**Patient refusal**				
	Yes	14 (78)	7.43 (2.16–14.85) *	2.11 (0.71–6.27)
	No	114 (32.0)	1	1
**Rise in BP/HTN**				
	Yes	15 (68.2)	4.49 (1.60–8.69) *	5.09 1.90–13.59) **
	No	114 (32.3)	1	1
**Blood not prepared**				
	Yes	8 (53.3)	2.33 (1.02–4.67) *	1.71 (0.71–4.08)
	No	115 (33.0)	1	1
**Department**				
	General surgery	39 (27.9)	1	1
	Gynecological surgery	13 (27.7)	0.33 (0.14–0.78) *	0.48 (0.20–1.15)
	Orthopedic surgery	33 (39.3)	0.33 (0.12–0.89) *	2.13 (1.09–4.14)
	Plastic surgery	15 (39.5)	0.56 (0.23–1.35) *	2.11 (0.87–5.16)
	Neurologic surgery	16 (37.2)	0.56 (0.20–1.53)*	1.56 (0.51–4.76)
	Ophthalmologic	14 (53.8)	0.51 (0.19–1.37)*	3.76 (1.41–10.0)**

*Significant at *p*-value < 0.25, **significant at *p*-value < 0.05, 1 = Reference, COR, crude odds ratio; AOR, adjusted odds ratio; BP, blood pressure; HTN, hypertension; OR, operation room.

## Discussion

In this study, the magnitude of elective surgery cancellation was 35%. The finding of this study is consistent with previous studies conducted in Tikur Anbessa specialized hospital (33.9%) and Asella Teaching and referral hospital (32.2%), Ethiopian (32.2%) (5, 13, 17). However, the cancellation rate of this study was higher than studies done in Uganda (28.8%), Sudan (20.0%), India (27.2%), and Jimma (23.0%) (8, 18–20). This disparity might be attributed to sociodemographic factors, sample size, research location, length, methodological discrepancies, and ineffective hospital administration practices.

In this study, Females were more likely than males in this research to cancel elective surgery. However, our findings contrast with the findings of a study done in Korea (21), which found that women were less likely to cancel elective surgery. The disparity might be attributed to females’ perceptions of elective surgery. It has been reported that female patients are more prone to suffer anxiety before surgery. Furthermore, the societal pressure imposed on males to act bravely and not show fragility may result in less probable cancellation of elective surgery) (12).

In this study, individuals with high blood pressure were more likely to cancel elective surgery than those with normal blood pressure. This was consistent with the findings of research done in Singapore (22). The probable explanation for this is that hypertension in the preoperative and postoperative period increases cardiovascular events, cerebrovascular events, hemorrhage, and mortality and should be treated before major elective non-cardiac surgery and cardiac surgery (23). For this reason, the medical professionals or health care team canceled the surgery to prevent further complications. It has been advised that elective surgery be postponed if the systolic blood pressure is 180 mmHg or higher, or the diastolic blood pressure is 110 mmHg or higher, due to the risk of complications (24).

In this study, patients in the ophthalmologic surgery department were more likely to cancel their elective surgery than patients in other departments. This might be owing to a shortage of senior specialties.

In this study, educational status and place of residence were associated with the cancellation of elective surgery, whereas they were not in the previous study. In our study, persons with no formal education were more likely to cancel elective surgery than those with formal education. This might be due to persons with no formal education being unable to receive, comprehend, and use health information to make good health decisions regarding elective surgery. Furthermore, lack of understanding, the patient’s overestimation of surgical mortality risk, and choice conflict may all lead to the cancellation of elective surgery.

Participants in this study who came from rural regions were more likely to cancel elective surgery than those who arrived from urban areas. The possible explanation is a shortage of transportation. Again the majority of Ethiopians live in rural regions and have low resources. Because of the enormous distances between the community and the nearest health institution, people living in rural regions confront significant obstacles (25). Terrain and a lack of road infrastructure can be an issue in some regions. Transportation costs may have also played a role in the of elective surgery.

### Strengths and limitations

To acquire full information on the research participants, both primary and secondary data were employed. This research additionally evaluates factors that were not previously evaluated. However, there are certain limitations to this study. This research included only individuals who were planned for elective surgery, but did not include mothers who gave birth through elective cesarean section. This study is also limited to public hospitals and excludes private hospitals, which results in an underestimation of research findings.

## Conclusion

This study found that about one-third of scheduled elective surgery was canceled. Being female, individuals with no formal education, and an increase in blood pressure, place of residence, and ophthalmologic surgery were significantly associated with the cancellation of elective surgery. Elective surgery cancellation may be reduced by adopting a few easy actions, and it can be decreased if patients with medical concerns are discovered early and referred for an aesthetics assessment shortly after their surgery is planned. In addition, hospital administrators and other stakeholders highly recommended proper scheduling, providing adequate information for the scheduled patient, fulfilling necessary operating room equipment, including blood cross-matched, and clear communication with the operating room team, particularly surgeons, to be available on their schedule, improving operating theater efficiency and proper counseling of the patients. Therefore, timely evidence-based reporting through the supervision team was advised to decrease cancellations.

## Data availability statement

The raw data supporting the conclusions of this article will be made available by the authors, without undue reservation.

## Ethics statement

The studies involving human participants were reviewed and approved by the Haramaya University Institutional Health Research Ethics Review Committee. Written informed consent to participate in this study was provided by the participants’ legal guardian/next of kin.

## Author contributions

DA, TW, MD, and SL contributed to the idea’s creation, development, revision of the proposal, data gathering, analysis, and report writing. AH, AS, and HK participated in data collection, analysis, and manuscript preparation. All authors reviewed and approved the final manuscript.

## References

[1] Solak A Pandza H Beciragic E Husic A Tursunovic I Djozic H. Elective case cancellation on the day of surgery at a general hospital in sarajevo: causes and possible solutions. *Mater Soc Med.* (2019) 31:49. 10.5455/msm.2019.31.49-52 31213956 PMC6511384

[2] Trentman T Mueller S Dormer CL Weinmeister KP. Day of surgery cancellations in a tertiary care hospital. *J Anesth Clin Res.* (2010) 1:2. 10.4172/2155-6148.1000109

[3] Gajida A Takai I Nuhu Y. Cancellations of elective surgical procedures performed at teaching hospital in northwest Nigeria. *J Med Trop.* (2016) 18:108–12. 10.4103/2276-7096.192244

[4] González-Arévalo A Gómez-Arnau J delaCruz F Marzal J Ramírez S Corral E Causes for cancellation of elective surgical procedures in a Spanish general hospital. *Anesthesia.* (2009) 64:487–93. 10.1111/j.1365-2044.2008.05852.x 19413817

[5] Ayele A Weldeyohannes M Tekalegn Y. Magnitude and reasons of surgical case cancellation at a specialized hospital in Ethiopia. *J Anesth Clin Res.* (2019) 10:2.

[6] Birhanu Y Endalamaw A Adu A. Root causes of elective surgical case cancellation in Ethiopia: a systematic review and meta-analysis. *Patient Saf Surg.* (2020) 14:46. 10.1186/s13037-020-00271-5 33298136 PMC7727239

[7] Desta M Manaye A Tefera A Worku A Wale A Mebrat A Incidence and causes of cancellations of elective operation on the intended day of surgery at a tertiary referral academic medical center in Ethiopia. *Patient Saf Surg.* (2018) 12:25. 10.1186/s13037-018-0171-3 30154916 PMC6109985

[8] Ismat M Mutwali MA Elkheir I Bur A Geregandi T. Cancellation of elective surgical operations in a teaching hospital at Khartoum Bahri, Sudan. *Sudan Med Monitor.* (2016) 11:45. 10.4103/1858-5000.185230

[9] Hori Y Nakayama A Sakamoto A. Surgery cancellations after entering the operating room. *Surgery.* (2016) 4:7. 10.1186/s40981-016-0066-1 29492435 PMC5813762

[10] Merga H Desalegn N. Prospective study of proportions and causes of cancellation of surgical operations at jimma university teaching hospital, Ethiopia. *Int J Anesth Res.* (2015) 3:87–90. 10.19070/2332-2780-1500022

[11] Karashi A Alsaif M Rashid F Alboosta H Almalki A. Cancellation of elective procedures on the day of surgery. *Bahrain Med Bull.* (2018) 158:1–4. 10.12816/0047464

[12] Rahimi A Maimaiti N Aghaei L. Reasons for surgery cancellation in a public hospital in Iran. *Malays J Public Health Med.* (2017) 17:29–34.

[13] Dedecho A Geda B Gonfa G. The magnitude of elective surgical patient cancellation and associated factors at assella teaching and referral hospital, Oromia Region, South East Ethiopia. *Clin Med Res.* (2020) 9:91. 10.11648/j.cmr.20200904.14

[14] Shiferaw A. Magnitude of case cancellation and associatedfactors among elective surgical cases in tikur anbesa specialized hospital. *Addis Ababa Ethiopia.* (2016) 1:1–36.

[15] González-Arévalo A. Causes for cancellation of elective surgical procedures. *J Assoc Anesth Great Britain Ireland.* (2009) 1:487–93. 10.1111/j.1365-2044.2008.05852.x 19413817

[16] Central Statistical Agency [CSA]. *The Federal Democratic Republic of Ethiopia Central Statistical Agency.* Longueuil, QC: CSA (2020).

[17] Harar Regional Health Bureau [HRHB]. *Unpublished Data Survey From Data Management Center*. Harar: Harar Regional Health Bureau (2021).

[18] Demilew B Yisak H Terefe A. Magnitude and causes of cancellation for elective surgical procedures in debre tabor general hospital: a cross-sectional study. *SAGE Open Med.* (2021) 9:20503121211003357. 10.1177/20503121211003357 33796304 PMC7975488

[19] Alfred O Felix O Emmanuel N Timothy M Galukande M. Prevalence and predictors of cancellation of elective surgical procedures at a tertiary hospital in uganda: across-sectional study. *Surg Res Pract.* (2020) 2020:1464098. 10.1155/2020/1464098 32258365 PMC7115171

[20] Nanjappa B Kabeer K Smile S. Elective surgical case cancellation-an audit. *Int J Cur Res Rev.* (2014) 6:19–23.

[21] Cho H Lee Y Lee S Kim J Kim T. Reasons for surgery cancellation in a general hospital: a 10-year study. *Int J Environ Health.* (2018) 16:7. 10.3390/ijerph16010007 30577514 PMC6338898

[22] Tan A Chiewa C Wang S Abdullah H Lam S Ong M Risk factors and reasons for cancellation within 24 h of scheduled elective surgery in an academic medical center: A cohort study. *Int J. Surg.* (2019) 66:72–8. 10.1016/j.ijsu.2019.04.009 31029875

[23] Soni S Shah R Chaggar R Saini E James J Elliot J Surgical cancellation rates due to peri-operative hypertension: implementation of multidisciplinary guidelines across primary and secondary care. *Aneshtesia.* (2021) 77:1314–20. 10.1111/anae.15084 32488972

[24] Hartle A McCormack T Heagerty A. The measurement of adult blood pressure and management of hypertension before elective surgery. *Anesthesia.* (2016) 71:327–36. 10.1111/anae.13348 26776052 PMC5066735

[25] Carlos V Sven Y Nyengo M Groen L Viste A. Transportation barriers to accessing health care for surgical conditions in Malawi a cross-sectional nationwide household survey. *BMC Public Health.* (2019) 19:264. 10.1186/s12889-019-6577-8 30836995 PMC6402149

